# The Impact of Surgical Margins and Adjuvant Radiotherapy in Patients with Undifferentiated Pleomorphic Sarcomas of the Extremities: A Single-Institutional Analysis of 192 Patients

**DOI:** 10.3390/cancers12020362

**Published:** 2020-02-05

**Authors:** Ole Goertz, Andreas Pieper, Leon von der Lohe, Ingo Stricker, Mehran Dadras, Björn Behr, Marcus Lehnhardt, Kamran Harati

**Affiliations:** 1 Department of Plastic Surgery, BG-University Hospital Bergmannsheil, Buerkle-de-la-Camp-Platz 1, D-44789 Bochum, Germany; 2 Department of Plastic Surgery, Martin-Luther Hospital, Caspar-Theyss-Strasse 27-29, D-14193 Berlin, Germany; 3 Institute of Pathology, Ruhr-University Bochum, Buerkle-de-la-Camp-Platz 1, D-44789 Bochum, Germany

**Keywords:** pleomorphic sarcoma, margins, survival, surgery, extremity

## Abstract

*Background:* Undifferentiated pleomorphic sarcomas are a frequent subtype within the heterogeneous group of soft tissue sarcomas. As the attainment of negative margins can be complicated at the extremities, we determined the prognostic significance of surgical margins in our patient population. *Methods:* We retrospectively determined the relationship between local recurrence-free survival (LRFS), overall survival (OS), and potential prognostic factors in 192 patients with UPS of the extremities who were suitable for surgical treatment in curative intent. The median follow-up time was 5.1 years. *Results:* The rates of LRFS and OS after 2 years were 75.7% and 87.2% in patients with R0-resected primary tumors and 49.1% and 81.8% in patients with R1/R2-status (LRFS: *p* = 0.013; OS: *p* = 0.001). Adjuvant radiotherapy significantly improved LRFS (5-year: 67.6% vs. 48.4%; *p* < 0.001) and OS (5-year: 82.8 vs. 61.8; *p* = 0.016). Both, negative margins and adjuvant radiotherapy were found to be independent prognostic factors in multivariate analysis. *Conclusions:* The data from this study could underscore the beneficial prognostic impact of negative margins on LRFS and OS. However, the width of negative margins seemed to be not relevant. Notably, adjuvant radiotherapy was not only able to decrease the risk of local failure but also improved OS in a significant manner.

## 1. Introduction

Soft tissue sarcomas are a heterogeneous group of rare, solid malignancies with a mesenchymal origin. They account for approximately 1% of all adult malignancies with an incidence rate of 6 per 100,000 inhabitants [1,2]. Soft tissue sarcomas (STS) comprise a group of more than 50 different histologic subtypes that differ in their oncological behavior and outcome [3]. Due to their rarity and heterogeneity, most studies regarding treatment outcomes in STS include all subtypes rather than focusing on specific subtypes. Undifferentiated pleomorphic sarcomas (UPS), previously termed malignant fibrous histiocytomas, are a frequent STS histotype accounting for 14% of all adult STS [4]. As a mesenchymal tumor, UPS can arise throughout the body, but 60% occur in the extremities [5].

Generally, STS can be subdivided into two genetic groups. Several STS subtypes harbor specific genetic alterations such as translocations or activating mutations whereas the majority of STS displays complex karyotypes [6,7,8]. Here, UPS belongs to those STS that show a high diversity of genomic alterations and oncogenic drivers. Recently published, the Cancer Genome Atlas Research Network characterized several alterations found in UPS samples [6]. Interestingly, genomic profiling revealed a notable molecular similarity between myxofibrosarcomas and UPS although myxofibrosarcomas exhibit a completely different clinical behavior and rarely metastasize. However, in both, UPS as well as MFS, copy number alterations frequently affected VGLL3 and YAP1 which encode TEAD cofactors in the Hippo signaling pathway enhancing the proliferative activity. To date, the Hippo signaling pathway has been shown to play a crucial role in the development of several different malignancies and, therefore, presents an attractive treatment target where inhibitors are currently researched extensively [9,10]. The low innate chemosensitivity of UPS towards the first-line chemotherapeutics Doxorubicin and Ifosfamide has promoted the development of novel treatment strategies including immunotherapeutic approaches [11]. Published in 2017, a multi-center phase 2 trial (SARC028) assessed the safety and activity of pembrolizumab, an anti-PD-1 antibody, in ten patients with advanced UPS and presented encouraging results [12]. Four of those ten UPS patients showed an objective response towards pembrolizumab. However, further studies are needed to determine the activity of anti-PD-1 antibodies in UPS patients with localized tumors as well as metastatic disease.

Within the heterogeneous group of STS, UPS belong to the more aggressive subtypes accompanied by a high risk of local recurrence and distant metastasis [13,14,15,16,17]. The reported 5-year survival rates range from 60% to 76% [5,18,19,20,21]. Those reporting studies have also determined several statistically significant indicators of survival which included tumor-related factors such as histologic grade, tumor size, and tumor depth. However, the prognostic impact of the surgical margin status remains controversial due to contradictory results. Furthermore, the influence of negative margin widths is still a subject of debate. However, it is important to define the optimal extent of surgery to ensure the best outcomes. Especially at the extremities, it is often difficult to attain wide clear margins due to functionally important structures. 

The purpose of this study was to gain more insight into the clinical behavior of extremity UPS and to determine relevant prognostic factors reviewing our institutional experience. We focused particularly on the prognostic impact of treatment-related factors such as surgical margins, clear margin widths, and adjuvant radiotherapy on disease outcome.

## 2. Patients and Methods

### 2.1. Patients 

Between 1997 and 2017, 203 patients with primary UPS of the extremities were treated surgically with curative intent at our institution. We excluded patients presenting with simultaneous metastases to maintain a homogenous study population. We restricted analyses to 192 participants with full information available on the outcome, histology, and surgical margins at the initial procedure. Patient follow-up was obtained from our database, medical records, and patient correspondence. The study was approved by the ethics committee of the Medical Faculty of the Ruhr-University Bochum with the registration number 15-5411. 

### 2.2. Treatment

The goal of surgical treatment for all patients was resection of the tumor with negative margins (R0) in curative intent. In epifascial lesions, a deep tumor-free margin of one fascial layer was intended. The indication for adjuvant radiation was given at the discretion of the interdisciplinary tumor board of either our institution or the referring institutions. Generally, all intermediate (G2) and high-grade (G3) tumors were recommended for further adjuvant radiation. After surgical resection, patients underwent a uniform follow-up regimen which included examinations (clinical, chest X-ray, contrast-enhanced MRIs) every three months in the first two years and every six months in the following three years, Several patients, especially individuals with recurrent tumors, had also consistent follow-up examinations beyond 5 years after tumor resection. 

### 2.3. Histopathological Classification 

All tumors were diagnosed and classified using the guidelines of the French Federation of Cancer Centers and the World Health Organization [22,23]. All resected tumors were histopathologically analyzed by an experienced pathologist of our institution. The tumor surfaces were dyed with ink and the pathological specimen was fixated with formalin. The quality of the surgical margins, as well as the width of the negative margins, were assessed by the same pathologist. 

### 2.4. Statistical Analysis

All patients were retrospectively analyzed regarding possible prognostic factors influencing survival. Overall survival (OS) was defined as the time period from the date of surgery for the primary disease to the date of death from any cause or the date of last follow-up assessment in living patients. Local recurrence-free survival (LRFS) has been defined as the time between the resection of the primary tumor and the first occurrence of local recurrence. In patients without local recurrence, the time period lasted from the date of the resection of the primary tumor to the date where the last follow-up assessment revealed no local recurrence. Survival analyses were performed via the Kaplan–Meier method including 95% confidence intervals (CIs) and log-rank test. To assess independent prognostic factors for OS and LRFS, we also have conducted multivariate analyses using the Cox proportional hazards model and the Wald test. Here, all prognostic factors that reached statistical significance in the univariate analyses with *p* < 0.05 were included. For the statistical analysis, we have used the statistical program Stata (Version 11.2, StataCorp, College Station, TX, USA). 

## 3. Results

### 3.1. Follow-Up and Patient Characteristics

The median follow-up time for the entire cohort was 5.1 years after diagnosis of the primary tumor. At the end of the follow-up time (cut-off date), 119 (62.0%) patients did not show any evidence of disease, In contrast, 11 patients (5.7%) were alive with the local recurrent disease and 13 (6.8%) were alive with distant metastases. Thirty-seven patients (19.3%) died because of their sarcoma disease and 12 patients (6.3%) died because of other causes (Table 1).

The median age at the time of primary diagnosis was 64.5 years (range 18.3–89.9). There were 106 (55.2%) male and 86 (44.8%) female patients. The distribution of the histologic grading was G1 in 8 cases (4.2%), G2 in 69 (35.9%) and G3 in 115 (59.9%). In total, 35 patients (18.2%) had at least one local recurrence, whereas 33 (17.2%) patients had two or more local recurrences. During the follow-up time, 52 (27.1%) patients developed distant metastases. 

### 3.2. Treatment Characteristics

Surgical treatment in one or more steps led to microscopically negative margins (R0) in 179 patients (93.2%), while 11 patients (5.7%) were left with microscopically positive margins (R1) and 2 (1.0%) with macroscopically positive margins (R2). In those patients with R1 or R2 margins, the tumors were too advanced and widespread for complete resection. 

Generally, all patients with G2 and G3 tumors were recommended to receive further adjuvant radiation in their home areas. A total of 103 patients (53.6%) received adjuvant radiotherapy after resection of the primary tumor, with a median overall dose of 60.0 Gray (range: 25.0–70.0). Furthermore, 12 patients (6.2%) received preoperative radiotherapy while 12 patients (6.2%) were treated with adjuvant chemotherapy and only 4 patients (2.1%) with neoadjuvant chemotherapy.

### 3.3. Univariate Analysis of LRFS

**Figure 1:**
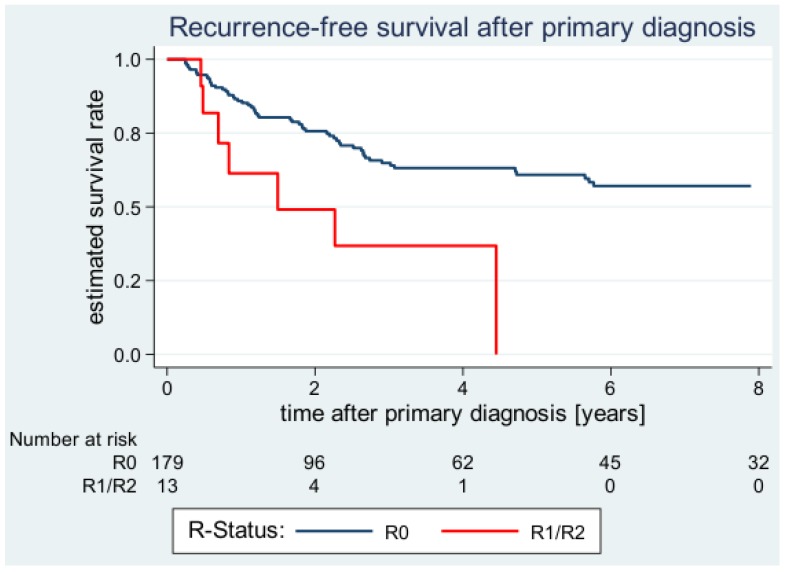
Estimated local recurrence-free survival (LRFS) curves after primary diagnosis according to margin status.

In the entire cohort, the 2-year and 5-year estimates of the LRFS rate were 74.1% (95% CI: 66.5–80.2) and 58.2% (95% CI: 49.3–66.0), respectively. The univariate analysis and comparison of patient- and tumor-related factors did not reveal any statistically significant findings. Patients under 60 years seemed to have a slightly better LRFS than the older patients, but the difference did not reach statistical significance (Table 2). Female patients tended to have a better prognosis than the male patients regarding local failure, although this distribution failed to gain statistical significance either. Patients with tumors larger than 5cm displayed a 5-LRFS of 49.0% (95% CI: 37.1–59.8) whereas patients with smaller tumors had a 5-year LRFS of 70.0% (95% CI: 56.6–80.0) (*p* = 0.141). Moreover, patients with deep, subfascial tumors and tumors arising in the upper extremities also tended to have worse LRFS rates, however, statistical differences were not significant. Interestingly, treatment-related factors such as surgical margin status and radiotherapy were the only factors to reach statistical significance in univariate analysis for LRFS (Table 2). Patients with R0 margins had a significantly more favorable LRFS than patients with R1 or R2 margins (2-year LRFS: R0 75.7 (67.9–81.8) vs. R1/R2 49.1% (16.7–75.3); *p* = 0.013) (Figure 1). Furthermore, we have also determined the impact of the negative margin widths within the subgroup of patients where tumors were resected with R0 margins. The quantitatively assessed negative margin widths were available for 140 of the 179 patients (78.2%) with R0-resected tumors. Here, the closest negative margin width was assessed as a potential treatment-related prognostic factor. However, univariate analysis could not reveal a significant difference between the patients groups: Patients with a negative margin width of ≤ 1mm had a 5-year LRFS of 64.1% (95% CI: 50.2–75.0) while patients with a negative margin width of > 5mm displayed a 5-year LRFS of 68.2 (95% CI: 39.5–85.4). Regarding adjuvant radiotherapy, patients treated with radiation had a significantly prolonged LRFS compared with patients whose primary tumors were not treated with postoperative radiation (5-year LRFS: 67.6% (55.0–77.3) vs. 48.4% (36.0–59.7); *p* < 0.001). 

### 3.4. Univariate Analysis of OS

The 2-year and 5-year estimate of the OS rate were 86.8% (95% CI: 80.5–91.2) and 73.0% (95% CI: 64.5–79.7). Patients under 60 years tended to have a better OS than older patients (5-year OS: 79.9% (65.4–88.8) vs. 69.0% (57.9–77.6); *p* = 0.192). In accordance with our findings for LRFS, male patients also tended to have a slightly worse outcome than female patients. Among the tumor-related factors, histologic grade, and tumor size and depth had a prognostic significance on OS in univariate analysis (Table 3). Patients with G2 tumors had more favorable prognoses than did patients with intermediate G3 lesions (5-year OS: G2 82.9% (68.7–91.1) vs. G3 67.9% (56.4–76.9); *p* = 0.017). In contrast to the findings for OS, tumor size and tumor depth reached statistical significance in univariate analysis. Primary tumors larger than 5 cm were associated with a significantly diminished outcome when compared with smaller tumors (5-year OS: 62.6% (50.9–72.2) vs. 89.1% (77.0–95.0); *p* = 0.002). Deep, subfascial localization also led to a significantly worse OS compared with epifascial lesions (5-year OS: 66.1% (55.6–74.6) vs. 91.7% (76.3–97.3); *p* = 0.003).

**Figure 2:**
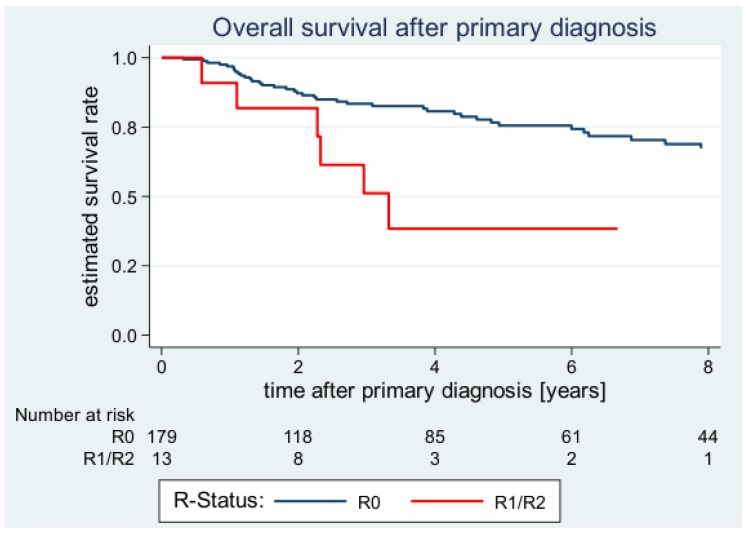
Estimated overall survival (OS) curves after primary diagnosis according to margin status.

Regarding the treatment-related factors, surgical margin status and adjuvant radiotherapy reached prognostic significance. Patients with R0 margins had a 2-year OS rate of 87.2% (95% CI: 80.6–91.6), whereas patients with R1/R2 margins presented with a 2-year OS rate of 81.8% (95% CI: 44.7–95.1) *p* = 0.001) (Figure 2). Within the R0 subgroup, the clear margin width did not influence OS significantly. However, there seemed to be a tendency that wide negative margin widths were associated with a more favorable prognosis: Patients with negative margin widths ≤1 mm had a 5-year OS rate of 76.8% (95% CI: 64.6–85.3), whereas patients with 1-5 mm had a rate of 83.2% (95% CI: 59.4–93.7) and patients with <5 mm 92.3% (95% CI: 56.6–98.9) (*p* = 0.070). Like findings for LRFS, adjuvant radiotherapy was associated with a significantly better OS (5-year OS: 82.8 (72.1–89.8) vs. 61.8 (48.2–72.8); *p* = 0.016).

### 3.5. Multivariate Analysis of LRFS

Surgical margin status and adjuvant radiotherapy were associated with *p* < 0.05 in the univariate analysis and, therefore, included in the Cox model to determine independent prognostic factors for LRFS (Table 4). Here, positive margins were associated with a significantly increased risk of local recurrence (Hazard ratio [HR]: 3.08 (1.48–6.39); *p* = 0.003), while adjuvant radiotherapy improved local control (HR: 0.41 [0.25–0.67); *p* < 0.001). We also have analyzed age and gender, but they failed to reach statistical significance in multivariate analysis.

### 3.6. Multivariate Analysis of OS

Significant and independent prognostic factors for the OS in the Cox model were histologic grade, tumor depth, surgical margin status, and adjuvant radiotherapy. Moreover, we have also included age and gender in the multivariate analysis. The HR for death was 2.65 (1.45–4.83) for G3 lesions (*p* = 0.001) and 4.65 (95% CI: 1.77–12.16) for subfascial tumors (*p* = 0.002). Regarding the treatment-related factors, positive margins were a strong negative predictive factor for OS (HR: 3.13 (1.54–6.38); *p* = 0.002). In accordance with our findings for the LRFS, adjuvant radiotherapy had a remarkably beneficial influence on OS with an HR of 0.50 [0.28–0.89] (*p* = 0.019). Notably, tumor size failed to reach statistical significance (*p* = 0.230) in multivariate analysis. Finally, age was found to be an independent prognostic factor of OS (*p* = 0.011)

## 4. Discussion

In the current study, we retrospectively analyzed the outcome of 192 patients with primary UPS of the extremities who underwent surgical treatment with curative intent. Most of the tumors displayed adverse biological features: They were high-grade (G3: 59.9%), subfascial localized (75.5%), and larger than 5 cm (64.0%). Although surgical treatment led to negative margins in 93.2% of all patients, 27.1% developed distant metastases during the median follow-up time of 5.1 years. The 5-year LRFS and OS rates were 58.2% and 73.0%, respectively. Notably, surgical margin status and adjuvant radiotherapy reached statistical significance as independent prognostic factors of both, LRFS and OS. Other independent predictive factors of OS were age, histologic grade, and tumor depth. 

To date, there have been a few retrospective analyses that assessed the outcome of patients with UPS (Table 5). Unfortunately, the results regarding the prognostic impact of surgical margins were inconsistent. In 2015, Dineen et al. from the MD Anderson Cancer Center in Houston determined the prognostic significance of surgical margins in 148 patients with sporadic and radiation-associated UPS of the extremities, the trunk and the head and neck area [5]. Although they could find an association between R0 margins and local control (*p* = 0.019), surgical margin status did not influence survival in their analysis (*p* = 0.501). Recently published, Vodanovich et al. presented the largest retrospective analysis for UPS including 266 patients with tumors in the extremities and the trunk [18]. Instead of the R classification Vodanovich et al. divided the quality of surgical margins into adequate margins including wide and radical resections and inadequate margins involving marginal and intralesional resections. In accordance with the findings of Dineen et al., inadequate margins had a statistically significant impact on local control but no on OS. Another large study was published by Le Doussal et al. in 1996 including 216 patients with extremity, trunk and head/neck UPS [21]. However, only R2-resections were found to be of prognostic significance while microscopic margins did not alter the outcome. In 2019, Kamat et al. presented a study involving 55 patients with lower-extremity UPS [19]. The 5-year disease-free survival rate for patients with positive margins was 33% compared with 63% for patients with negative margins (*p* = 0.03). Vice versa, they did not find an association between surgical margins and LRFS. 

In our series, patients with microscopic negative margins had a significantly better LRFS and more favorable OS than patients with positive margins. To define the optimal surgical margin, we also analyzed the prognostic influence of the closest negative margin widths in 140 of the 179 patients (78.2%) with R0-resected tumors. Here, we could not detect a significant beneficial influence of wide negative margin widths. However, patients with wide negative margin widths tended to have a better OS: Patients categorized with negative margin widths >5 mm had a 5-year OS rate of 92.3% whereas patients with negative margin widths ≤1 mm displayed a 5-year OS-rate of 76.8%. This finding would provide a more aggressive surgical approach in order to attain wider negative margins. Past studies of our institution which have determined the influence of negative margin widths in STS, in general, could not establish a relationship between margin widths and outcome [24,25]. However, analyses of STS subsets such as angiosarcoma and dermatofibrosarcoma protuberans revealed that margin widths may indeed have an impact on specific entities [26,27,28]. Therefore, it is crucial to analyze the entities separately and to assess the prognostic significance of treatment-related factors for every histologic subset specifically. Unfortunately, the low number of cases worldwide impede those efforts and decision-making is based on studies mixing all STS subsets together. 

Adjuvant radiotherapy was another important treatment-related factor that had a remarkable impact on LRFS and OS in our series. Most of the studies focusing on UPS demonstrated that adjuvant radiotherapy improved significantly local control (Table 5). However, none of the aforementioned studies could detect a beneficial impact of radiotherapy on OS. To date, there has been only one randomized, prospective trial that has assessed the impact of adjuvant radiation in STS patients with curative resectable tumors [29]. Here, Beane et al. analyzed the outcome of 141 patients with extremity STS who underwent limb-sparing surgery. 70 of these 141 patients received adjuvant external beam radiation therapy while 71 patients were only treated with limb-sparing surgery. Although the 10-year OS rate was 77% for the non-radiated patients and 82% for the radiated patients, this statistical distribution did not gain statistical significance in their analysis (*p* = 0.22). However, the reason may be due to the relatively low proportion of patients with high-grade STS in their prospective study (29%). In a SEER-based analysis, Koshy et al. determined the effect of radiotherapy on OS among patients with STS of the extremities who underwent limb-sparing surgery [30]. Notably, 59% of all patients had high-grade lesions and they could show that radiotherapy improved OS in patients with high-grade STS significantly. This is in line with our findings and may be caused by the high proportion of high-grade tumors (60.9%) in our patient population.

Finally, the reservation must be made that our series only included patients with primary UPS that were suitable for further surgical treatment with curative intent. We excluded patients with concurrent metastases at the time of primary diagnosis or patients who were treated in palliative intent. Hence, our findings are only applicable to the selected group of patients where further surgical treatment was possible and not to all patients with UPS. This implies a study selection bias that must be acknowledged. 

## 5. Conclusions

In conclusion, this study could underscore the independent prognostic significance of surgical margins for LRFS and OS. It is noteworthy that only the quality of surgical margins, but not the negative margin width had a prognostic influence. Moreover, adjuvant radiotherapy had a similarly strong impact on local control as well as survival which may be due to the high proportion of high-grade tumors in our series. With respect to the currently available data from the present and previous studies on UPS, treatment efforts should aim at limb-sparing resections when feasible with negative margins involving radiotherapy to ensure optimal local control and survival. 

## Figures and Tables

**Table 1:** Patient and disease characteristics.

Characteristic	*N*	% of Total
Total	192	
Age (years)		
≤60	72	37.5
>60	120	62.5
Sex		
Female	86	44.8
Male	106	55.2
Tumor size		
≤5 cm	69	36.0
>5 cm	123	64.0
Tumor depth		
Epifascial	47	24.5
Subfascial	145	75.5
Tumor site		
Upper extremity	66	34.4
Lower extremity	126	65.6
Grading		
G1	8	4.2
G2	69	35.9
G3	115	59.9
Margin status (Primary tumor)		
R0	179	93.2
R1	11	5.7
R2	2	1.0
Adjuvant radiotherapy (Primary tumor)		
No	89	46.4
Yes	103	53.6
Neoadjuvant radiotherapy (Primary tumor)		
No	180	93.8
Yes	12	6.2
Adjuvant chemotherapy (Primary tumor)		
No	180	93.8
Yes	12	6.2
Neoadjuvant chemotherapy (Primary tumor)		
No	188	97.9
Yes	4	2.1
Status		
No evidence of disease	119	62.0
Alive with local recurrent disease	11	5.7
Alive with distant metastases	13	6.8
Died due to UPS	37	19.3
Died due to other causes	12	6.3

**Table 2:** Results of the univariate analyses to determine factors predictive of LRFS.

Characteristic	*N*	No. of Local Recurrence	1-Year LRFS (95%-CI)	2-Year LRFS (95%-CI)	5-Year LRFS (95%-CI)	*p* (Log-Rank)
All patients	192	68	84.4 (78.1–89.0)	74.1 (66.5–80.2)	58.2 (49.3–66.0)	
Age (years)						
≤60	72	25	90.8 (80.7–95.8)	72.0 (58.7–81.6)	62.4 (48.4–73.5)	
>60	120	43	80.5 (71.6–86.8)	76.0 (66.4–83.1)	55.3 (43.4–65.7)	0.371
Sex						
Female	86	27	88.2 (78.5–93.7)	81.9 (70.8–89.1)	63.3 (49.6–74.2)	
Male	106	41	81.5 (72.3–88.0)	67.9 (57.0–76.6)	53.9 (41.9–64.5)	0.194
Tumor size						
≤5 cm	69	22	84.0 (72.3–91.1)	75.4 (62.5–84.4)	70.0 (56.6–80.0)	
>5 cm	123	46	84.6 (76.3–90.1)	72.9 (62.8–80.7)	49.0 (37.1–59.8)	0.141
Tumor depth						
Epifascial	47	17	86.6 (72.6–93.8)	76.5 (60.7–86.7)	65.4 (48.3–78.0)	
Subfascial	145	51	83.6 (75.9–89.0)	73.2 (64.1–80.3)	55.5 (44.9–64.8)	0.708
Tumor site						
Upper extremity	66	31	84.3 (72.8–91.2)	73.6 (60.4–83.0)	49.8 (35.1–62.8)	
Lower extremity	126	37	84.4 (76.1–90.0)	74.3 (64.5–81.8)	63.6 (52.6–72.7)	0.169
Grading						
G1	8	4	87.5 (38.7–98.1)	87.5 (38.7–98.1)	72.9 (27.6–92.5)	
G2	69	31	84.8 (73.5–91.5)	74.7 (62.1–83.7)	55.0 (41.3–66.7)	
G3	115	33	83.9 (75.0–89.8)	72.6 (62.0–80.7)	60.4 (47.9–70.8)	0.799 *
Margin status (Primary tumor)						
R0	179	61	85.9 (79.5–90.4)	75.7 (67.9–81.8)	60.9 (51.8–68.7)	
R1/R2	13	7	61.4 (26.6–83.5)	49.1 (16.7–75.3)	(–)	0.013
Negative margin width						
≤1 mm	88	26	90.2 (81.3–95.0)	78.2 (66.7–86.1)	64.1 (50.2–75.0)	
>1 mm and ≤5 mm	36	5	96.3 (76.5–99.5)	86.9 (64.3–95.6)	76.6 (52.1–89.7)	
>5 mm	16	5	75.0 (46.3–89.8)	75.0 (46.3–89.8)	68.2 (39.5–85.4)	0.272 *
Adjuvant radiotherapy (Primary tumor)						
No	89	44	79.3 (68.8–86.6)	66.5 (54.7–75.9)	48.4 (36.0–59.7)	
Yes	103	24	89.0 (80.5–93.9)	81.1 (70.8–88.0)	67.6 (55.0–77.3)	<0.001
Neoadjuvant radiotherapy (Primary tumor)						
No	180	63	83.9 (77.2–88.7)	74.9 (67.1–81.1)	58.3 (49.1–66.5)	
Yes	12	5	91.7 (53.9–98.8)	66.7 (33.7–86.0)	57.1 (25.4–79.6)	0.953
Adjuvant chemotherapy (Primary tumor)						
No	180	62	80.5 (68.2–88.5)	71.1 (57.6–81.0)	63.2 (48.1–75.0)	
Yes	12	6	86.3 (78.3–91.5)	75.3 (65.5–82.7)	56.0 (44.7–65.8)	0.656
Neoadjuvant chemotherapy (Primary tumor)						
No	188	66	84.0 (77.6–88.8)	74.1 (66.4–80.3)	58.4 (49.4–66.4)	
Yes	4	2	100 (–)	75.0 (12.8–96.1)	50.0 (5.8–84.5)	-

* global log-rank test for trend of survivor functions. LRFS, local recurrence-free survival; CI, confidence interval.

**Table 3:** Results of the univariate analyses to determine factors predictive of OS.

Characteristic	*N*	No. of Deaths	1-Year OS (95%-CI)	2-Year OS (95%-CI)	5-Year OS (95%-CI)	*P* (Log-Rank)
All patients	192	50	96.5 (92.4–98.4)	86.8 (80.5–91.2)	73.0 (64.5–79.7)	
Age (years)						
≤60	72	16	100 (–)	91.2 (80.1–96.3)	79.9 (65.4–88.8)	
>60	120	34	94.4 (87.9–97.4)	84.1 (75.4–90.0)	69.0 (57.9–77.6)	0.192
Sex						
Female	86	20	100 (–)	92.5 (82.9–96.8)	71.9 (57.9–81.9)	
Male	106	30	93.9 (86.9–97.2)	82.5 (72.9–88.9)	74.1 (63.1–82.3)	0.274
Tumor size						
≤5 cm	69	11	98.5 (89.6–99.8)	93.4 (83.2–97.5)	89.1 (77.0–95.0)	
>5 cm	123	39	95.4 (89.2–98.0)	82.9 (73.8–89.0)	62.6 (50.9–72.2)	0.002
Tumor depth						
Epifascial	47	5	100 (–)	97.5 (83.5–99.6)	91.7 (76.3–97.3)	
Subfascial	145	45	95.3 (89.8–97.8)	83.0 (74.9–88.7)	66.1 (55.6–74.6)	0.003
Tumor site						
Upper extremity	66	20	96.7 (87.4–99.2)	86.4 (74.6–93.0)	69.9 (55.2–80.6)	
Lower extremity	126	30	96.4 (90.8–98.6)	87.1 (78.7–92.3)	75.0 (64.1–82.9)	0.561
Grading						
G1	8	4	100 (–)	87.5 (38.7–98.1)	60.0 (19.5–85.2)	
G2	69	14	100 (–)	100 (–)	82.9 (68.7–91.1)	
G3	115	32	93.9 (86.9–97.2)	77.8 (67.6–85.1)	67.9 (56.4–76.9)	0.017 *
Margin status (Primary tumor)						
R0	179	44	96.9 (92.6–98.7)	87.2 (80.6–91.6)	75.5 (66.9–82.2)	
R1/R2	13	6	90.9 (50.8–98.7)	81.8 (44.7–95.1)	38.4 (10.3–66.8)	0.001
Negative margin width						
≤1 mm	88	22	96.3 (89.1–98.8)	84.0 (73.5–90.6)	76.8 (64.6–85.3)	
>1 mm and ≤5 mm	36	4	93.2 (75.4–98.3)	89.6 (71.1–96.5)	83.2 (59.4–93.7)	
>5 mm	16	1	100 (–)	100 (–)	92.3 (56.6–98.9)	0.070 *
Adjuvant radiotherapy (Primary tumor)						
No	89	31	96.3 (88.9–98.8)	81.7 (71.0–88.8)	61.8 (48.2–72.8)	
Yes	103	19	96.7 (90.2–98.9)	91.7 (83.3–96.0)	82.8 (72.1–89.8)	0.016
Neoadjuvant radiotherapy (Primary tumor)						
No	180	46	96.2 (91.8–98.3)	86.5 (79.8–91.1)	73.8 (65.0–80.7)	
Yes	12	4	100 (–)	91.7 (53.9–98.8)	66.7 (33.7–86.0)	0.997
Adjuvant chemotherapy (Primary tumor)						
No	180	43	96.4 (90.7–98.6)	86.6 (78.3–91.8)	73.9 (63.3–81.8)	
Yes	12	7	96.7 (87.4–99.2)	87.4 (75.3–93.8)	72.0 (56.7–82.7)	0.885
Neoadjuvant chemotherapy (Primary tumor)						
No	188	48	96.4 (92.2–98.4)	87.2 (80.8–91.5)	73.8 (65.2–80.5)	
Yes	4	2	100 (–)	75.0 (12.8–96.1)	50.0 (5.8–84.5)	-

* global log-rank test for trend of survivor functions. OS, overall survival; CI, confidence interval.

**Table 4:** Results of multivariate analysis on LRFS and OS according to the Cox proportional hazard model.

Category (Reference)	LRFS		OS	
	HR (95% CI)	*p*	HR (95% CI)	*p*
Depth: subfascial (vs. epifascial)	-	-	4.65 (1.77–12.16)	0.002
Size: >5 cm (vs. ≤5 cm)	-	-	1.63 (0.73–3.63)	0.230
Grade: G3 (vs. G1)	-	-	2.65 (1.45–4.83)	0.001
Margin status: R1/R2 (vs. R0)	3.08 (1.48–6.39)	0.003	3.13 (1.54–6.38)	0.002
Adjuvant radiation: Yes (vs. no)	0.41 (0.25–0.67)	<0.001	0.50 (0.28–0.89)	0.019
Sex: Male (vs. female)	1.41 (0.86–2.33)	0.175	1.04 (0.53–2.02)	0.916
Age: >60 (vs. ≤60)	1.23 (0.76–2.00)	0.396	2.32 (1.21–4.44)	0.011

LRFS, local recurrence-free survival; OS, overall survival; MFS; CI, confidence interval. Factors that did not reach statistical significance (*p* < 0.05) in the univariate analysis were not included in the multivariate analysis.

**Table 5:** Overview of retrospective analyses on undifferentiated pleomorphic sarcomas (UPS).

Author (Year)	*N*	Site	Median FU (Years)	5-LRFS (%)	5-OS (%)	Independent Prognostic Effect of Microscopic Margins on		Independent Prognostic Effect of Adjuvant Radiotherapy on	
						LRFS	OS/DSS	LRFS	OS/DSS
Present study	192	Extremity	5.1	58	73	+	+	+	+
Kamat (2019)	55	Lower Extremity	4.5	60	68	−	+	+	−
Vasileios (2012)	61	Extremity	4.3	NA	67	−	−	−	−
Dineen (2015)	148 ^a^	Extremity, Trunk, Head/Neck	NA	75	76 ^b^	+	−	−	−
Vodanovich (2018)	266	Extremity, Trunk, Head/Neck	7.0	85	60	+	−	−	−
Le Doussal (1996)	216	Extremity, Trunk, Head/Neck	3.5	63	70 ^b^	−	−	+	−

FU, follow-up; 5-LRFS, local recurrence-free survival rate at 5 years; 5-OS, overall survival at 5 years; 5-DSS, disease-specific survival at 5 years; NA, data not available. ^a^ matched cohort analysis; ^b^ value for 5-year DSS.

## References

[1] Saltus C.W., Calingaert B., Candrilli S., Lorenzo M., D’Yachkova Y., Otto T., Wagner U., Kaye J.A. (2018). Epidemiology of Adult Soft-Tissue Sarcomas in Germany. Sarcoma.

[2] Jemal A., Siegel R., Ward E., Murray T., Xu J., Thun M.J. (2007). Cancer statistics, 2007. CA Cancer J. Clin..

[3] Katz D., Palmerini E., Pollack S.M. (2018). More Than 50 Subtypes of Soft Tissue Sarcoma: Paving the Path for Histology-Driven Treatments. ASCO.

[4] Brennan M.F., Antonescu C.R., Moraco N., Singer S. (2014). Lessons learned from the study of 10,000 patients with soft tissue sarcoma. Ann. Surg..

[5] Dineen S.P., Roland C.L., Feig R., May C., Zhou S., Demicco E., Sannaa G.A., Ingram D., Wang W.L., Ravi V. (2015). Radiation-Associated Undifferentiated Pleomorphic Sarcoma is Associated with Worse Clinical Outcomes than Sporadic Lesions. Ann. Surg. Oncol..

[6] Lazar A.J., McLellan M.D., Bailey M.H., Miller C.A., Appelbaum E.L., Cordes M.G., Fronick C.C., Fulton L.A., Fulton R.S., Mardis E.R. (2017). Comprehensive and Integrated Genomic Characterization of Adult Soft Tissue Sarcomas. Cell.

[7] Taylor B.S., Barretina J., Maki R.G., Antonescu C.R., Singer S., Ladanyi M. (2011). Advances in sarcoma genomics and new therapeutic targets. Nat. Rev. Cancer.

[8] Barretina J., Taylor B.S., Banerji S., Ramos A.H., Lagos-Quintana M., Decarolis P.L., Shah K., Socci N.D., Weir B.A., Ho A. (2010). Subtype-specific genomic alterations define new targets for soft-tissue sarcoma therapy. Nat. Genet..

[9] Helias-Rodzewicz Z., Perot G., Chibon F., Ferreira C., Lagarde P., Terrier P., Coindre J.M., Aurias A. (2010). YAP1 and VGLL3, encoding two cofactors of TEAD transcription factors, are amplified and overexpressed in a subset of soft tissue sarcomas. Genes Chromosome Cancer.

[10] Kakiuchi-Kiyota S., Schutten M.M., Zhong Y., Crawford J.J., Dey A. (2019). Safety Considerations in the Development of Hippo Pathway Inhibitors in Cancers. Front. Cell Dev. Biol..

[11] Kawaguchi K., Igarashi K., Kiyuna T., Miyake K., Miyake M., Murakami T., Chmielowski B., Nelson S.D., Russell T.A., Dry S.M. (2018). Individualized doxorubicin sensitivity testing of undifferentiated soft tissue sarcoma (USTS) in a patient-derived orthotopic xenograft (PDOX) model demonstrates large differences between patients. Cell Cycle.

[12] Tawbi H.A., Burgess M., Bolejack V., Van Tine B.A., Schuetze S.M., Hu J., D’Angelo S., Attia S., Riedel R.F., Priebat D.A. (2017). Pembrolizumab in advanced soft-tissue sarcoma and bone sarcoma (SARC028): A multicentre, two-cohort, single-arm, open-label, phase 2 trial. Lancet Oncol..

[13] Stojadinovic A., Leung D.H., Hoos A., Jaques D.P., Lewis J.J., Brennan M.F. (2002). Analysis of the prognostic significance of microscopic margins in 2084 localized primary adult soft tissue sarcomas. Ann. Surg..

[14] Zagars G.K., Ballo M.T., Pisters P.W., Pollock R.E., Patel S.R., Benjamin R.S., Evans H.L. (2003). Prognostic factors for patients with localized soft-tissue sarcoma treated with conservation surgery and radiation therapy: An analysis of 1225 patients. Cancer.

[15] Lehnhardt M., Daigeler A., Homann H.H., Schwaiberger V., Goertz O., Kuhnen C., Steinau H.U. (2009). MFH revisited: Outcome after surgical treatment of undifferentiated pleomorphic or not otherwise specified (NOS) sarcomas of the extremities—An analysis of 140 patients. Langenbecks Arch. Surg..

[16] Callegaro D., Miceli R., Bonvalot S., Ferguson P., Strauss D.C., Levy A., Griffin A., Hayes A.J., Stacchiotti S., Pechoux C.L. (2016). Development and external validation of two nomograms to predict overall survival and occurrence of distant metastases in adults after surgical resection of localised soft-tissue sarcomas of the extremities: A retrospective analysis. Lancet Oncol..

[17] Pisters P.W., Leung D.H., Woodruff J., Shi W., Brennan M.F. (1996). Analysis of prognostic factors in 1041 patients with localized soft tissue sarcomas of the extremities. J. Clin. Oncol..

[18] Vodanovich D.A., Spelman T., May D., Slavin J., Choong P.F.M. (2019). Predicting the prognosis of undifferentiated pleomorphic soft tissue sarcoma: A 20-year experience of 266 cases. ANZ J. Surg..

[19] Kamat N.V., Million L., Yao D.H., Donaldson S.S., Mohler D.G., van de Rijn M., Avedian R.S., Kapp D.S., Ganjoo K.N. (2019). The Outcome of Patients with Localized Undifferentiated Pleomorphic Sarcoma of the Lower Extremity Treated at Stanford University. Am. J. Clin. Oncol..

[20] Vasileios K.A., Eward W.C., Brigman B.E. (2012). Surgical treatment and prognosis in patients with high-grade soft tissue malignant fibrous histiocytoma of the extremities. Arch. Orthop. Trauma Surg..

[21] Le Doussal V., Coindre J.M., Leroux A., Hacene K., Terrier P., Bui N.B., Bonichon F., Collin F., Mandard A.M., Contesso G. (1996). Prognostic factors for patients with localized primary malignant fibrous histiocytoma: A multicenter study of 216 patients with multivariate analysis. Cancer.

[22] Coindre J.M. (2006). Grading of soft tissue sarcomas: Review and update. Arch. Pathol. Lab. Med..

[23] Fletcher C.D. (2014). The evolving classification of soft tissue tumours–An update based on the new 2013 WHO classification. Histopathology.

[24] Harati K., Kolbenschlag J., Bohm J., Niggemann H., Joneidi-Jafari H., Stricker I., Lehnhardt M., Daigeler A. (2018). Long-term outcomes of patients with soft tissue sarcoma of the chest wall: Analysis of the prognostic significance of microscopic margins. Oncol. Lett..

[25] Harati K., Goertz O., Pieper A., Daigeler A., Joneidi-Jafari H., Niggemann H., Stricker I., Lehnhardt M. (2017). Soft Tissue Sarcomas of the Extremities: Surgical Margins Can Be Close as Long as the Resected Tumor Has No Ink on It. Oncologist.

[26] Harati K., Daigeler A., Goertz O., Bohm J., Lange K., Stricker I., Kolbenschlag J., Lehnhardt M. (2016). Primary and Secondary Soft Tissue Angiosarcomas: Prognostic Significance of Surgical Margins in 43 Patients. Anticancer Res..

[27] Lehnhardt M., Bohm J., Hirsch T., Behr B., Daigeler A., Harati K. (2017). Radiation-induced angiosarcoma of the breast. HaMiPla.

[28] Harati K., Lange K., Goertz O., Lahmer A., Kapalschinski N., Stricker I., Lehnhardt M., Daigeler A. (2017). A single-institutional review of 68 patients with dermatofibrosarcoma protuberans: Wide re-excision after inadequate previous surgery results in a high rate of local control. World J. Surg. Oncol..

[29] Beane J.D., Yang J.C., White D., Steinberg S.M., Rosenberg S.A., Rudloff U. (2014). Efficacy of adjuvant radiation therapy in the treatment of soft tissue sarcoma of the extremity: 20-year follow-up of a randomized prospective trial. Ann. Surg. Oncol..

[30] Koshy M., Rich S.E., Mohiuddin M.M. (2010). Improved survival with radiation therapy in high-grade soft tissue sarcomas of the extremities: A SEER analysis. IJROBP.

